# Oral Surgery

**Published:** 1888-03

**Authors:** R. C. Brewster


### ORAL SURGERY.

BY R. C. BREWSTER, M. D. S.

READ BEFORE THE BROOKLYN DENTAL SOCIETY.

Of all the departments within the wide scope of our profession, I know of none more deserving our attention than Oral Surgery, or one which is so largely neglected. In selecting a subject for writing, I have determined to narrate some cases that I have seen in hospitals and dispensaries, and discuss them from a pathological standpoint. Such patients, however, as one meets there, are always unsatisfactory, as they are likely to disappear as soon as made comfortable. In the event of a return of the disease, they are quite as likely to go to some other similar institution; these people are very properly called "rounders." Then, too, the physical standard of such patients is far below those in private practice, caused largely by poor dwelling places and insufficient food and clothing. However, sufficient can be seen to obtain a general knowledge of almost every case that presents itself.

The first case of importance that came under my observation was that of a German girl, about fourteen years of age, fairly well developed, suffering intensely from a swollen face which completely closed the right eye, and also from the extreme heat of the August weather. On examination, I found the crown of the right superior second molar broken off, the other teeth being apparently good. I extracted the roots, but no pus followed. On closer examination I found the first bicuspid opaque and extracted it, and a copious flow of thin, dark, fetid pus shot out as if from great pressure. The discharge continued so long that I told her to come again the following day, instructing her to keep the cavity open and allow a free discharge. She came at the appointed time, and I was able to make a thorough examination, and found the cavity opened into the antrum, which was unusually large, due to the bulging out of the anterior wall from the extreme pressure of the fermenting fluid within, and whichever way I turned the probe it grated as against the side of a brick, but I could not pass it into the nasal cavity.

No history of the case could be obtained, other than that she had suffered from toothache for about three months. I conducted the patient to the surgical department, where a consultation was held, and they decided to operate by making a sufficient opening and to remove the necrosed bone by a bur, to all of which her parents consented. On further deliberation wiser counsel prevailed, and it was deemed prudent to wait until more favorable conditions could be obtained and the patient's extremely debilitated condition could be improved, and also for cooler weather. In the meantime I was to have charge and do what I could for her by the use of aromatic sulphuric acid. I accordingly instructed her how to prepare a fifty per cent. aqueous solution of the acid, provided her with an ordinary rubber syringe, and told her to syringe it out thoroughly twice a day, first with a good supply of tepid water and afterward with the syringe twice filled with the acid, and also to keep the opening tightly packed with absorbent cotton to enlarge it, and to return each week for further instruction. I also gave her for constitutional treatment syr. iod. ferri., ten drops, t. i. d. and plenty of good nutritious food, such as beef, soup, milk, eggs, etc., from the diet dispensary.

I saw her about once a week during the next three months, and at the end of that time she had no need of any surgical operation. The flow of pus had entirely stopped, and the opening was closed. The bulging out of the superior maxillary bone remained the same, and does to this day.

Another case was that of an Italian woman, about forty years of age, who had a swelling on the left side of her face, slight, however, as compared with the other, and who came to ask for some medicine to take a bad taste out of her mouth. With a hectic flush upon her cheek and a deep cough, there was no difficulty in diagnosing hers to be a case of consumption. She was neat and clean, with well developed features, but the odor from her month was very bad. She also complained of toothache on the left side of her face, and I found, on examination, that the teeth from the superior centrals to the second molars were either badly decayed or broken off, and the gums greatly swollen. After considerable persuasion she consented to have them extracted, which resulted in extensive hemorrhage, mixed with pus, the character of which could not at that time be distinguished.

After the hemorrhage had sufficiently subsided, she was told to come the next day, when the diagnosis was very simple. Through the sockets of the eye tooth and first and second bicuspids, the handle of an excavator readily passed to the floor of the orbit, with the same sense of roughness that always denotes necrosis. There was still a copious flow of sanious pus, and after thoroughly evacuating the antrum and syringing it out with a four per cent. aqueous solution of carbolic acid, she was told to come the following week, that being the earliest time she had at her disposal. She was given a sufficient amount of aromatic sulphuric acid, diluted one-half with water, a syringe and cotton, and told to syringe it twice a day, and to pack it with cotton after each dressing, and no doubt she did, for at the end of a week she returned, and complained that her face was very much worse; that she had done exactly as she had been told, and had put a piece of cotton in the hole twice a day until she could no longer get any in! She had forgotten to remove each piece that was put in the time before, and had been simply adding piece after piece, twice a day, until no more would enter. Consequently, the use of the syringe had been of no effect. I found, on examination, that the antrum was packed very closely with cotton, and it required a long time to remove it, the odor at the same time being dreadful. The disturbance was now very greatly increased from the pressure of the cotton against the floor of the orbit, producing orbital cellulitis, and the patient was sure she would lose the eye. Such, however, did not prove to be the case, for at the end of another week, with thorough cleansing and proper drainage, all inflammation around the eye had entirely disappeared. I also noticed considerable exfoliation of the alveolus, which was easily removed with an excavator. At the end of another week a large portion of the superior maxillary bone had also exfoliated. It was the anterior inferior portion, from the canine fossa to the anterior nasal spine, including a portion of the palatine process. Some difficulty was experienced in removing this piece, as the gums had to be laid open, and the edges were very ragged and sharp, which caused considerable hemorrhage. However, after diligent effort it was finally removed, and the hemorrhage stopped by compression.

I gave her the aromatic sulphuric acid dressing, with syringe, etc., and after some trouble in teaching her how to use it, soon began to see its good effects. In about another month the discharge had entirely ceased, and no trace of necrosis could be found. The opening at the canine fossa was about one quarter of an inch in diameter, and the passage from the antrum into the nasal cavity about the same.

I now made for her a partial plate which so restored the fullness of the lip that the deformity was scarcely noticeable, and aside from the annoyance of cleaning the cavity after each meal, she had no trouble. For constitutional treatment I gave her syp. iod. ferri., ten drops three times a day, and cod-liver oil.

I am fully aware of the popular prejudice against aromatic sulphuric acid for such cases, but I have found that patients will use that when they will use nothing else, and the results have been entirely satisfactory. No one will attempt to dispute the advisability of this treatment over an operation with the knife in these two cases.

Another case was that of a puny, sickly boy of four years, who had to be carried in his mother's arms to the dispensary, and who had the same aromatic sulphuric acid treatment for necrosis in his right inferior maxillary, which exfoliated a piece of bone an inch and one half long and one quarter inch wide, and the treatment resulted in complete recovery in three months. An operation with the knife could have made no better result, and, to say the least, would have been injudicious. The character of the discharge of the first two cases, thin, dark and fetid, I have always found the result of necrosis.

